# Dissecting intratumoral myeloid cell plasticity by single cell RNA‐seq

**DOI:** 10.1002/cam4.2113

**Published:** 2019-04-29

**Authors:** Qianqian Song, Gregory A. Hawkins, Leonard Wudel, Ping‐Chieh Chou, Elizabeth Forbes, Ashok K. Pullikuth, Liang Liu, Guangxu Jin, Lou Craddock, Umit Topaloglu, Gregory Kucera, Stacey O’Neill, Edward A. Levine, Peiqing Sun, Kounosuke Watabe, Yong Lu, Martha A. Alexander‐Miller, Boris Pasche, Lance D. Miller, Wei Zhang

**Affiliations:** ^1^ Center for Cancer Genomics and Precision Oncology Wake Forest Baptist Comprehensive Cancer Center, Wake Forest Baptist Medical Center Winston Salem North Carolina; ^2^ Department of Cancer Biology Wake Forest School of Medicine Winston Salem North Carolina; ^3^ Department of Biochemistry Wake Forest School of Medicine Winston Salem North Carolina; ^4^ Department of Surgery Wake Forest School of Medicine Winston Salem North Carolina; ^5^ Department of Pathology Wake Forest School of Medicine Winston Salem North Carolina; ^6^ Department of Immunology and Microbiology Wake Forest School of Medicine Winston Salem North Carolina

**Keywords:** intercellular interaction, monocyte‐to‐M2 differentiation, non‐small cell lung cancer (NSCLC), single‐cell RNA sequencing (scRNA‐seq), trajectory analysis

## Abstract

Tumor‐infiltrating myeloid cells are the most abundant leukocyte population within tumors. Molecular cues from the tumor microenvironment promote the differentiation of immature myeloid cells toward an immunosuppressive phenotype. However, the in situ dynamics of the transcriptional reprogramming underlying this process are poorly understood. Therefore, we applied single cell RNA‐seq (scRNA‐seq) to computationally investigate the cellular composition and transcriptional dynamics of tumor and adjacent normal tissues from 4 early‐stage non‐small cell lung cancer (NSCLC) patients. Our scRNA‐seq analyses identified 11 485 cells that varied in identity and gene expression traits between normal and tumor tissues. Among these, myeloid cell populations exhibited the most diverse changes between tumor and normal tissues, consistent with tumor‐mediated reprogramming. Through trajectory analysis, we identified a differentiation path from CD14+ monocytes to M2 macrophages (monocyte‐to‐M2). This differentiation path was reproducible across patients, accompanied by increased expression of genes (eg, *MRC1*/*CD206*, *MSR1*/*CD204*, *PPARG*, *TREM2*) with significantly enriched functions (Oxidative phosphorylation and P53 pathway) and decreased expression of genes (eg, *CXCL2*, *IL1B*) with significantly enriched functions (*TNF‐α* signaling via *NF‐κB* and inflammatory response). Our analysis further identified a co‐regulatory network implicating upstream transcription factors (*JUN*, *NFKBIA*) in monocyte‐to‐M2 differentiation, and activated ligand‐receptor interactions (eg, *SFTPA1*‐*TLR2*, *ICAM1*‐*ITGAM*) suggesting intratumoral mechanisms whereby epithelial cells stimulate monocyte‐to‐M2 differentiation. Overall, our study identified the prevalent monocyte‐to‐M2 differentiation in NSCLC, accompanied by an intricate transcriptional reprogramming mediated by specific transcriptional activators and intercellular crosstalk involving ligand‐receptor interactions.

**Abbreviations**
AT1 and AT2: alternative cell fate 1 and cell fate 2
CNV: copy number variation
DC: dendritic cell
M1: type I macrophages
M2: type II macrophages
NK: natural killer cell
P1: Patient1
P2: Patient 2
P3: Patient 3
P4: Patient 4
PCA: principal component analysis
ROS: reactive oxygen species
ScRNA‐seq: single cell RNA sequencing
Th: T‐helper cell
TME: tumor microenvironment
TTPSR: tumor tissue and pathology shared resource

## 1 INTRODUCTION

Lung cancer is one of the leading causes of cancer‐related death worldwide.^1^ Non‐small cell lung cancer (NSCLC) is the most common histologic subtype of lung cancer and represents 85% of newly diagnosed cases.^2^ Five‐year survival rates for early stage NSCLC are only 83% and 71% for those patients with pathologic stage IA and IB respectively, and a dismal 50% for stage II NSCLC.^3^ For early stage NSCLC patients, surgical resection remains the mainstay of therapy; however, despite margin negative resections, tumor recurrence occurs in about 40% of patients. Adjuvant chemotherapy offers minimal benefit for stage II NSCLC patients.^4^ In a recent trial, neoadjuvant immunotherapy generated efficacious responses in 9 of 20 early‐stage NSCLC cases^5^ through revitalizing durable immune response. Although new immunomodulatory and immunotherapy treatment strategies for NSCLC patients are of potential gain, prudent use requires a detailed understanding of the immune cell landscape that develops specifically in response to tumor cues.

Tumors grow in unison with an intricate network of infiltrating immune cells that impact tumor progression in diverse ways. Under activating conditions, effector CD8 + T‐cells and CD4 + T‐helper cells elicit antitumor immunity and suppress tumor growth.^6^ *IFN‐*γ and *TNF‐α* secreted by T‐helper cells are primarily responsible for potentiating the cytotoxic T‐cell response.^7^ By contrast, T‐cell immunity can be abolished through T cell exhaustion induced by immunosuppressive cytokines (eg, *IL‐10*, *TGF*‐*β*) secreted by cancer cells or CD4 + regulatory T cells, or by antigen persistence.^8^, ^9^ Similarly, B cells with regulatory functions can promote tumor progression by secretion of immunosuppressive cytokines and conversion of T cells to regulatory T cells, while other B cell types can facilitate anti‐tumor immunity through Th1 cytokine secretion, antigen presentation, or antibody‐dependent mechanisms.^10^

Myeloid cells, such as tumor‐associated monocytes and macrophages, also possess positive and negative roles in tumor development and progression. Known for their functional and molecular plasticity, monocytes are recruited to the tumor site by tumor‐secreted chemo‐attractants (eg, *CCL2*,^11^, ^12^ *S100A8*/*S100A9*), and rapidly reprogrammed by the tumor microenvironment (TME).^11^ Depending on the cytokine milieu, monocytes can differentiate into M1‐macrophages associated with phagocytic activity and Th1‐promoting cytokine production (eg, *IL‐12*, *IL‐23*, *TNF‐α*); dendritic cells that promote anti‐tumor immunity through antigen presentation and cytokine production^13^, ^14^; or pro‐tumorigenic M2‐macrophages that promote immune suppression (eg, *IL‐10*, *TGF‐β*, *PD‐L1*, *ARG‐1*), angiogenesis (eg, *VEGF*, *ADM*, *PDGF*), and metastasis (via cancer cell migration, invasion, and extravasation).^15^

Consequently, a detailed understanding of how components of the immune system are transcriptionally reprogrammed in response to tumor cues will benefit the design of therapeutic strategies for NSCLC treatment. While microarray and RNA‐seq‐based mRNA deconvolution analysis of bulk tumor tissues has provided novel insight into the cellular composition of NSCLC and other cancers,^16^ single cell RNA‐sequencing (scRNA‐seq) of dissociated tumor tissues enables the analysis of transcriptional heterogeneity at single‐cell resolution,^17^ reconstruction of evolutionary lineages,^18^ and modeling of cellular cross‐talk within the TME.^19^ Recently scRNA‐seq was used to investigate the heterogeneity and diversity of tumor‐infiltrating myeloid cell types in lung adenocarcinoma lesions.^20^ However, little was inferred regarding the developmental dynamics of these myeloid cell populations.

In this context, we performed scRNA‐seq in both adjacent normal and tumor tissues from 4 early‐stage NSCLC patients. We observed a remarkable change of immune cells, especially myeloid cells, between adjacent normal and tumor tissues in each patient. To uncover the underlying molecular basis for such changes, we used the Monocle 2 method to construct the differentiation trajectory of myeloid cells through ordering cells coordinately from the adjacent normal to tumor tissues. This analysis shed light on the monocyte‐to‐M2 differentiation as a prevalent trajectory and revealed dysregulated genes likely involved in this process. Moreover, we identified upstream regulators and potential crosstalk signals that mediate the monocyte‐to‐M2 differentiation.

## 2 MATERIALS AND METHODS

### 2.1 Human sample acquisition

In our study, samples from 4 patients diagnosed with non‐small cell lung cancer (NSCLC) were included. Fresh remnant tumor and adjacent normal tissues were collected at the time of elective curative resection by the Tumor Tissue and Pathology Shared Resource (TTPSR) of the Wake Forest Baptist Medical Center Comprehensive Cancer Center (WFBMC‐CCC). Collections by the TTPSR adhere to Institutional Review Board approved procedures (Advanced Tumor Bank protocol CCCWFU 01403, TTPSR collections IRB BG04‐104 which also allows for the use of de‐identified protected health information along with the tissue samples). Acquisition of de‐identified samples from the TTPSR for single cell isolation and research use was in accordance with approved IRB protocol 00048977. Available clinical characteristics of these patients, managed through a HIPAA‐compliant database, are summarized in Tables S1 and S2.

### 2.2 Tissue dissociation and single cell RNA sequencing

Fresh tumor and adjacent normal tissue samples were collected by the TTPSR into tissue storage medium (Miltenyi) and stored at 4°C. Within 24 hours, tissues were processed to single‐cell suspensions using the human tumor dissociation kit and GentleMACS protocols with subsequent RBC removal by negative selection using CD235a microbeads (Miltenyi, following recommended procedures). Recovered cell numbers were determined by trypan blue exclusion using an automated counter (LUNA II). Cells were viably frozen in 10% Hybri‐Max DMSO (Sigma‐Aldrich): 90% heat‐inactivated FBS by cooling in isopropanol at −1°C per minute at −80°C overnight, and subsequently stored under liquid nitrogen vapor. In preparation for scRNA‐seq, cells were thawed and washed according to the demonstrated protocol for human PBMCs (10× Genomics).

All scRNA‐seq procedures were performed by the Cancer Genomics Shared Resource (CGSR) of the WFBMC‐CCC. Viable cells (mean 66.5 ± 8.6%, n = 8) in suspensions averaging 677 ± 198 cell/µl were loaded into wells of a 10× Chromium single cell capture chip targeting a cell recovery rate of 2000 ‐ 4000 cells. Single‐cell gel beads in emulsion (GEMs) were created on a Chromium Single Cell Controller and scRNA‐seq libraries were prepared using the Chromium Single Cell 3’ Library and Gel Bead kit according to the manufacturer's protocol (10× Genomics). Sequencing libraries were loaded at 1.3 PM on an Illumina NextSeq500 with High Output 150 cycle kit (Illumina) for paired‐end sequencing using the following read length: 26 bp Read1, 8 bp i7 Index, 0 bp i5 Index, and 98 bp Read2.

### 2.3 Single cell RNA sequencing data processing

The Cell Ranger Single Cell Software Suite v.2.0.1 was used to perform sample de‐multiplexing, alignment, filtering, and UMI (ie, universal molecular identifier) counting (https://support.10xgenomics.com/single-cell-gene expression/software/pipelines/latest/what‐is‐cell‐ranger). The data for each respective subpopulation were aggregated for direct comparison of single cell transcriptomes. The complete spreadsheet of the sequencing metrics is presented in the Supplemental Information (Table S4). A total of 11 813 single cells consisting of the 4 paired samples were captured, with the number of cells recovered per channel ranging from 369 to 2502. The mean reads per cell varied from 42 577 and 297 451 with median Unique Molecular Indexes of 2695 to 15 758 per cell. Low‐quality cells were discarded if the cell number with expressed genes was smaller than 200. Cells were also removed if their proportions of mitochondrial gene expression were larger than 40%. Final cell number of 4 paired patients is 11 485. The variations caused by tissue locations, that is, adjacent normal and tumor, are removed through utilizing the “merge_gbm” and “concatenate_gene_bc_matrices” functions.

### 2.4 CNV estimation

Copy number variations (CNVs) were inferred using the CONICS tool^21^ (COpy‐Number analysis In single‐Cell RNA‐Sequencing; https://github.com/diazlab/CONICS/), which is designed to infer large‐scale CNVs from single‐cell RNA‐seq data. Using nonmalignant cells (bottom of figure) to establish the “no‐CNV” threshold, the average expression (of chromosomally ordered genes) within a sliding window of 200 genes allows for the statistical discovery of CNV candidate regions (CNVCR) on a per cell basis. By fitting a Gaussian mixture model to the CNVCRs, a FDR‐corrected *P*‐value (from chi‐square test) can be obtained that indicates the significance of a candidate CNVCR. Cells containing 1 or more significant CNVCRs are identified as putative cancer cells (Figure S2).

### 2.5 PCA‐based unsupervised clustering

We used the Seurat toolkit^22^ to perform principal component analysis (PCA) and t‐distributed stochastic neighbor embedding (t‐SNE) analysis. Only cells that expressed more than 200 genes were considered, and only genes that expressed in at least 5 single cells were included. The data matrices were imported into R and the Seurat R package version 2.0.0. Data were read into R as a counts matrix and log transformed. To account for the possibility of individual cell complexity driving cluster separation, we employed Seurat's “RegressOut” function to reduce the contribution of both the number of UMI's and the percent mito. Variable genes were then determined using 4 cutoffs, including the top/bottom cutoff on x‐axis and y‐axis, respectively, x.high.cutoff = 8, x.low.cutoff = 0, y.high.cutoff = Inf, y.cutoff = 0. These cutoffs were placed on the mean variability to specify and select the variable genes. These variable genes were then used for subsequent PCA for each separate individual. For T‐distributed Stochastic Neighbor Embedding (tSNE) projection and clustering analysis, we used the first 30 principal components, which were determined using the standard deviations of the principal components visualized by PCA Elbow plot in Seurat. Then we used the feature plot function to highlight expression of known marker genes for CD14+ monocyte (*CD14*, *FCGR3A*) and CD1c+ DC or CD141+ DC (*CD1C*, *THBD*) to identify the specific cell clusters (Figure S3‐S6).

### 2.6 Single cell annotation

To identify the cell type identity for each cell cluster, we developed the cell‐type score through benchmarking of the expression levels of specific markers in 1 cluster compared to all other clusters^23^ (Table S3). Specifically, we assume the total number of clusters as *N* and the number of cell types as T. Given a set of genes ($G_{t}$ ,1≤t≤T) that reflect 1 specific cell type, that is, the markers of cell type (t), we define the enrich score, $C_{i}\left(G_{t}\right)$ (1≤t≤T,1≤i≤N) for the cell cluster $C_{i}$, to quantify the enrichment level of genes $G_{t}$ in the cell cluster $C_{i}$.

$$C_{i}\left(G_{t}\right)=\sum_{\left(1\leq j\leq N)\cap (j\neq i\right)}C_{\mathrm{ij}}\left(G_{t}\right)$$

In the above equation, $C_{\mathrm{ij}}\left(G_{t}\right)$ measures the difference between cell cluster i and (1≤i≠j≤N), which is defined by the sum of $\log_{2}$ fold changes of the genes with adjusted *P*‐value less than 0.05 in $G_{t}$, that is,

$$C_{\mathrm{ij}}\left(G_{t}\right)=\sum_{ge\in G_{t}\cap P_{\mathrm{ij}}\left(ge\right)0.05}{\mathrm{FC}}_{\mathrm{ij}}\left(ge\right)$$

As above, ${\mathrm{FC}}_{\mathrm{ij}}\left(ge\right)$ and $P_{\mathrm{ij}}\left(ge\right)$ refer to the $\log_{2}$ fold changes and adjusted *P*‐value of gene ge in comparing the cell cluster $C_{i}$ and $C_{j}$. The $\log_{2}$ fold changes and adjusted *P*‐value were computed using the R function “edgeR”, which was designed for single cell differential analysis based on the negative binomial distributions. In the NSCLC specimens, we identified fibroblasts, CD14+ monocytes, CD14− monocytes, M1 macrophages, M2 macrophages, CD1c+ dendritic cell (DC), CD141+ DC, natural killer cells, B cells, T‐helper cells, CD8+ T cells, and other T cells (Figure S2‐S5).

### 2.7 Trajectory analysis

The Monocle2^18^ method was used to construct the single cell trajectories in order to reveal the tumor‐reprogramming processes in myeloid cells. As human CD141+ DC and CD1c+ DC share similar transcriptional profiles with the murine CD103+ DC and CD11b+ DC respectively, and are thought to represent their human counterparts,^24^, ^25^ we involved the CD1c+ DC but excluded the CD141+ DC in the trajectory analysis, due to their differences in the lineage development. We used differentially expressed genes identified by Seurat to sort cells in the spatial‐temporal differentiation order. Cells in less differentiation type, that is, monocytes from the adjacent normal tissues, informed us of the start point of the pseudo‐time in the first round of “orderCells”. We then set this state as the root_state argument and called “orderCells” again. “DDRTree” was applied to reduce dimensions and the visualization functions “plot_cell_trajectory” or “plot_complex_cell_trajectory” were used to plot the minimum spanning tree on cells. Significance of differentially expressed genes was calculated with an approximate likelihood ratio test (Monocle2 differentialGeneTest() function) of the full model “~state” cells against the reduced model “~1”. For the dynamically expressed genes, the full model “~sm.ns(Pseudotime)” was tested against the reduced model of no pseudotime dependence. In both cases, *P* values were normalized using the Benjamini‐Hochberg, selecting statistically significant genes with *P* < 0.005 and FDR < 0.05.

### 2.8 Functional analysis

#### 2.8.1 Hallmark collection

The Hallmark gene set collection used for the functional analysis is downloaded from the Molecular Signatures Database (MSigDB),^26^ a widely used and comprehensive database. Each hallmark in this collection consists of a “refined” gene set that conveys a specific biological state or process and displays coherent expression. The hallmarks effectively summarize most of the relevant information of the original founder sets and, by reducing both variation and redundancy, provide more refined and concise inputs for gene set enrichment analysis.

#### 2.8.2 Pathway database

Reactome (http://www.reactome.org) is a manually curated open‐data resource of human pathways and reactions, an archive of biological processes and a tool for discovering potential functions. Gene sets derived from the Reactome^27^ and KEGG^28^ pathway database were downloaded from the MSigDB Collections.

#### 2.8.3 Enrichment test

Functional enrichment based on the above respective databases was assessed by hypergeometric test, which was used to identify a priori‐defined gene sets that showed statistically significant differences between two given clusters. The test was performed by the clusterProfiler package.^29^ We further corrected the test *P*‐values by the Benjamini‐Hochberg and less than 0.05 was considered as statistically significant.

### 2.9 Cell‐cell interactions

We identified putative interactions between any pair of cell types based on expression of a receptor by cells from one cell type and expression of an interacting ligand by cells from the other cell type: whenever a ligand transcript is “expressed” by a single cell from cell type A and the interacting receptor transcript is “expressed” by a single cell from cell type B, we define it as one potential ligand‐receptor interaction between A and B. The set of potential receptor‐ligand interactions were obtained from the IUPHAR (International Union of Pharmacology) and the connectome^30^ databases, which included totally 790 ligands, 711 receptors, and 2695 ligand‐receptor interactions.

## 3 RESULTS

### 3.1 The immunological composition of NSCLC varies across patients and tumor/normal pairs

**Figure 1:**
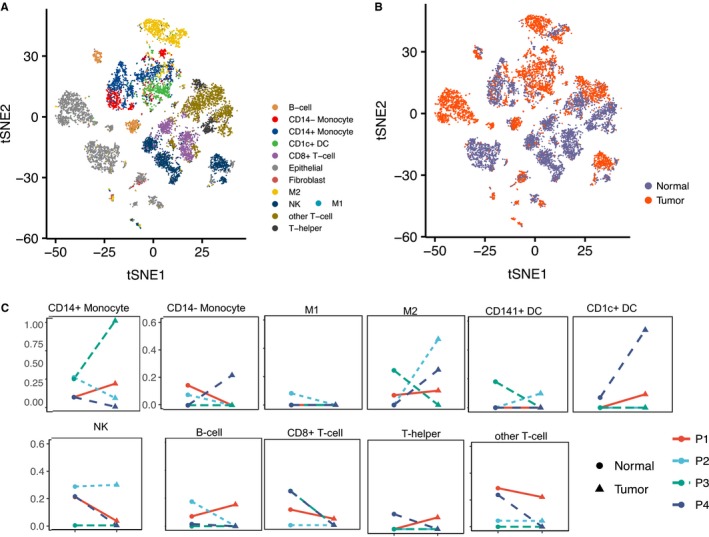
The immunological composition of NSCLC varies across patients and tumor/normal pairs. Cell clusters resolved by PCA‐based t‐distributed stochastic neighbor embedding (t‐SNE) are shown in aggregate analysis of paired tumor/normal samples, and identified by cell type in (A), and tumor versus normal tissue of origin in (B). (C) Cell type‐specific plots illustrate the tumor‐to tumor heterogeneity in the irregularity of change of immune cell abundance (*Y* axis, percentage) between matched normal and tumor tissues. Patients are indicated by colored lines, tissue type by circle or triangle

Matched tumor (T) and adjacent normal (N) tissues from 4 early‐stage NSCLC patients (designated as P1‐P4, Table S1) were analyzed by scRNA‐seq analysis. High‐quality transcriptomic data were obtained for 11 485 cells (Table S2). PCA‐based clustering was used to evaluate cell transcriptional heterogeneity. Immune cell type‐specific gene markers described in^23^, ^31^ were used to annotate immune cell populations (Table S3), and CNV analysis confirmed the identity of malignant epithelial cells (Figure S1‐S5). In aggregate analysis, 12 distinct cell populations were identified (Figure 1A) that showed substantial variation in abundance between tumor and normal tissues (Figure 1B).

While most immune cell types were consistently identified across patient specimens, their relative proportions varied from patient to patient (Figure S6) and showed no consistent pattern between matched T and N tissues (Figure 1C). Relative to normal tissue, proportions of CD8 + T cells and NK cells decreased or remained constant in tumors. Large proportional differences between T and N were observed for monocytes, M2 macrophages, and DCs. Overall we found a large degree of variation in the immune composition among the 4 tumors, which agreed with RNA‐seq (bulk tumor) deconvolution analysis of immune cells in TCGA NSCLC tumors (Figure S7). Similar immune phenotypic variability has been reported in multiple cancer types.^20^, ^32^, ^33^

### 3.2 Myeloid cell reprogramming

**Figure 2:**
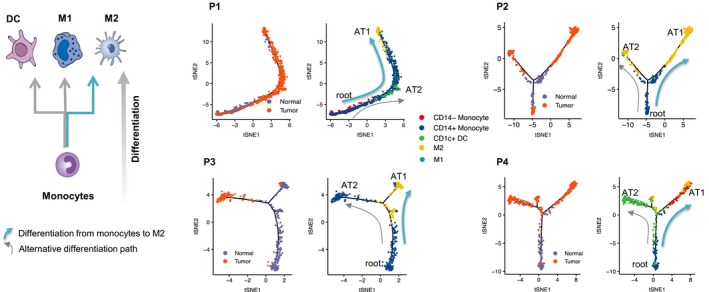
Myeloid cell reprogramming in each patient. Left panel shows the differentiation paths involved in the myeloid cells reprogramming. Right panel includes the plots delineating the myeloid cell reprogramming trajectory for each patient (P1‐P4). Cells on the trajectories are aligned in the order of differentiation (the arrow shape), representing the gradual transition from initial state to cell fate state. The trajectory on the left of each plot shows the tissue source of cells located on the trajectory (cyan, adjacent normal tissue; orange, tumor tissue). The trajectory on the right of each plot shows the cells colored by cell types (eg, blue, CD14+ monocytes; yellow, M2 macrophages)

We observed large T‐N proportional differences in myeloid cell types in all 4 tumors (Figure 1C). Myeloid cell reprogramming, a common feature of the TME, is known to be a continuous differentiation process.^34^ Depending on specific cues from the TME, monocytes can differentiate into inflammatory macrophages (M1 macrophages), monocyte‐derived DCs (CD1c+ or CD141+ DC) with anti‐tumor immune functions, or alternatively activated macrophages (M2 macrophages) with immunosuppressive properties (Figure 2).

To quantitatively track myeloid reprogramming between adjacent normal and tumor states, we applied the Monocle2 trajectory analysis method^18^, ^35^ to the myeloid cells from each patient (Figure 2; P1‐P4). Each T‐N trajectory is composed of a lower “root”, referring to the monocytes from adjacent normal tissues, and “branches” (annotated as AT1 or AT2) that reflect the monocyte differentiation toward M1‐like macrophage, M2‐like macrophage, or dendritic cell fates.

In P1 (Figure 2, P1), the trajectory analysis revealed a gradual transition from the root monocyte state to the AT1 cell fate of M2 macrophages. Monocytes from T tissue were identified as existing in an intermediate state, suggesting their reprogramming from the monocyte root in N tissue (Figure 2; Figure S8). For P1, most cells undergoing differentiation appeared to follow the AT1 fate and become M2 macrophages. Only a few cells went through the AT2 fate becoming the CD1c+ DC.

The remaining patients exhibited similar differentiation paths from N monocytes to T M2 macrophages but with notable exceptions (Figure 2, P2‐P4). Some N monocytes were observed as intermediate state in P2, while in the other 3 patients, N monocytes only appeared as the root state. Branched trajectories with diverse alternative differentiation outcomes were observed in P2, P3, and P4. P2 exhibited a second path from monocyte to M1 macrophage. P3 displayed an alternative monocyte differentiation path and P4 had an alternative path from monocytes to CD1c+ DC. Overall, the differentiation from monocyte to M2 macrophage appeared to be the predominant path in myeloid reprogramming.

### 3.3 Systematic myeloid cell reprogramming across patients

**Figure 3:**
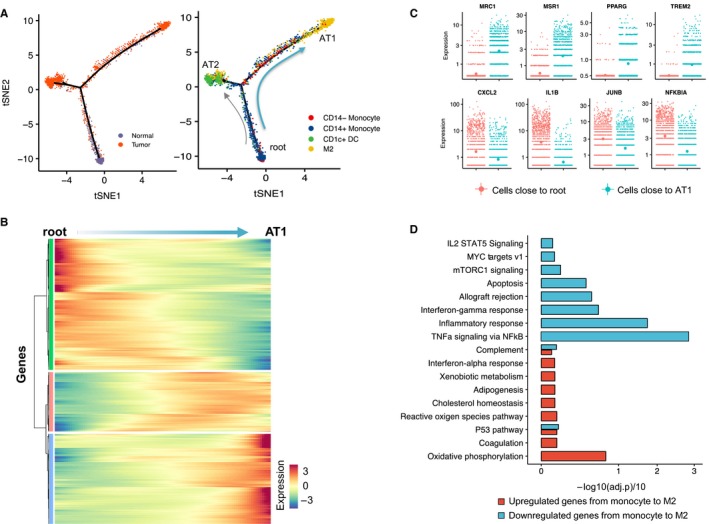
Systematic myeloid cell reprogramming across patients. (A). Cells on the trajectories are aligned in the order of differentiation (the arrow shape), representing the gradual transition from initial state to cell fate state. The left trajectory shows the tissue source of cells (cyan, adjacent normal tissue; orange, tumor tissue). The right trajectory shows the cells colored by cell types (eg, blue, CD14+ monocytes; yellow, M2 macrophages). (B) Heatmap shows the gradual up‐ and downregulated expression of genes during the monocyte‐to‐M2 differentiation. Genes (row) are clustered to 3 groups for better visualization and cells (column) are ordered according to the monocyte‐to‐M2 differentiation path (ie, from root to AT1); (C). Scatter plots show the expression of cell differentiation markers in individual cells involved in the monocyte‐to‐M2 differentiation. The y‐axis represents the relative gene expression while the *x*‐axis represents the monocyte‐to‐M2 differentiation. Each dot in the scatter plot represents the gene expression (lg(counts + 1)) of each cell. (D). Significantly, enriched terms in the Hallmark collection (*y*‐axis) are shown in bar plots based on the gradually up (red) and down‐regulated (cyan) genes in the monocyte‐to‐M2 differentiation. The *x*‐axis represents ‐lg(adj.p)/10, which is calculated by the enrichment test (see Methods)

To identify genes associated with myeloid cell reprogramming, we merged the myeloid cells of 3 patients and applied trajectory analysis. Only P3 was excluded due to its small cell number. In agreement with the individual patient trajectories, the global trajectory defines the lower root initial state as being comprised of monocytes primarily from normal tissue (Figure 3A), and the cell fate branches as comprising predominantly of tumor‐derived differentiated cells (M2 macrophages or CD1c+ DC). Focusing on the branch from root to AT1 (monocyte‐to‐M2), the trajectory reflected sequential gene expression changes or “transition state” genes. Transition state genes were identified as incrementally upregulated or downregulated from root to AT1 (Figure 3B). Upregulated genes included differentiation markers (eg, *MRC1*/*CD206*, *MSR1*/*CD204*, *PPARG,* and *TREM2*) known to be associated with M2 macrophage polarization. Downregulated genes included the pro‐inflammatory cytokines (*CXCL2* and *IL1B*) and transcription factors (*JUNB* and *NFKBIA*), representative of repressed genes in monocyte differentiation to the M2 phenotype (Figure 3C). Interestingly, distributed along the monocyte‐to‐M2 transition but not showing root‐ or branch‐level enrichment, cells are observed with the expression of the markers for monocytic myeloid‐derived suppressor cells (M‐MDSC; including *IL10*, *CD14,* and *VEGFA*) (Figure S9A) as well as markers for polymorphonuclear myeloid‐derived suppressor cells (PMN‐MDSC; including *IL6*, *OLR1,* and *TGFB1*) (Figure S9B). These observations are consistent with evidence that M‐MDSCs and PMN‐MDSCs are molecularly distinct from M1 and M2 macrophages,^14^ and may reflect transitional states related to M2 cell differentiation.

Next, we examined the enriched functions associated with these transitional genes in the GSEA Gene Hallmark Collection^36^ (Figure 3D). We identified enrichment associated with oxidative phosphorylation and P53 pathway in the upregulated genes, whereas *TNF‐α *signaling via *NF‐κB* and inflammatory response categories were significantly associated with the downregulated genes. Additionally, we analyzed the genes for enrichment of GO function terms (Figure S8). The GO terms with antigen processing and presentation functions were shown to be enriched in the gradually upregulated genes, while the nuclear‐transcribed mRNA catabolic process was enriched in the gradually downregulated genes.

### 3.4 Co‐regulatory network in the monocyte‐to‐M2 differentiation

**Figure 4:**
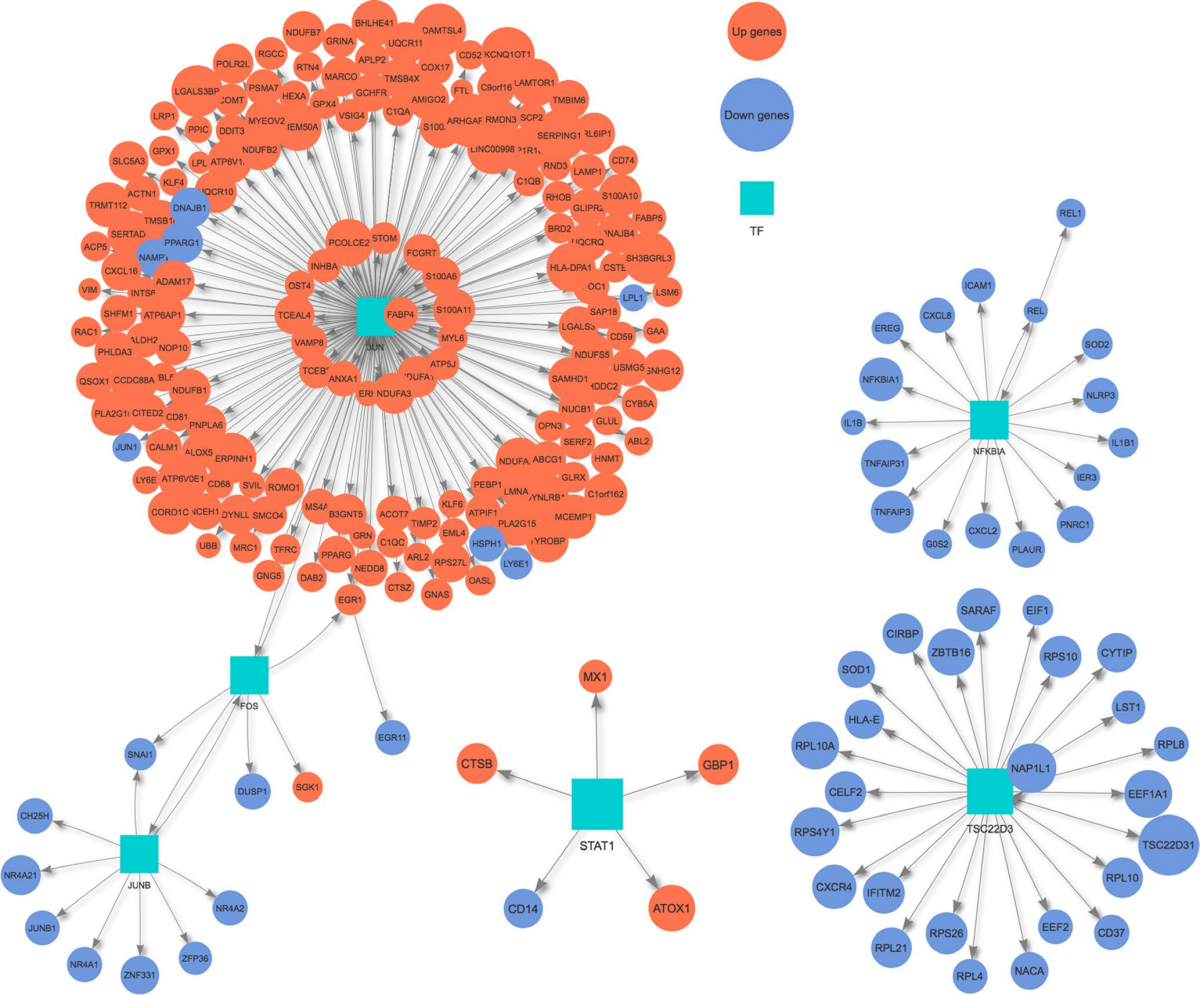
Co‐regulatory network in the monocyte‐to‐M2 differentiation. Transcriptional regulatory network involved in the monocyte‐to‐M2 differentiation. Transcriptional regulators are shown as nodes (square) with connected genes (circle). Orange color represents the upregulated genes whereas light blue represents the downregulated genes in the monocyte‐to‐M2 differentiation

To identify putative transcriptional regulators of the monocyte‐to‐M2 transition‐state genes, we utilized the Ingenuity Pathway Analysis (IPA)^37^ Upstream Regulator Tool and the GENIE3^38^ method. Significant co‐expressed regulatory networks associated with the upregulated and downregulated genes were identified (Figure 4). *JUN* was observed as the transcriptional hub mediating the upregulated genes, whereas *NFKBIA* (one of the downregulated genes) was identified as a significant mediator of the downregulated genes, suggesting that elevation of *JUN* and repression of *NFKBIA* underlie monocyte‐to‐M2 differentiation. Notably, *JUN* has immunosuppressive roles in macrophages and the phosphorylation of *c‐Jun* is acquired by the immunosuppressive protumorigenic macrophage phenotype,^39^ while the overexpression of *IκBα *(*NFKBIA*) inhibits *NF‐kB* activation in tumor‐associated macrophages and is associated with reduced tumor formation in hepatocellular carcinoma.^40^

### 3.5 Inference of intercellular interactions that mediate the monocyte‐to‐M2 differentiation

The differentiation from monocytes to M2 macrophages prompted us to consider intercellular interactions, such as ligand‐receptor signaling, in the TME. Through the IPA upstream analysis, we identified upstream receptors associated with the transitional genes. We inferred putative intercellular interactions based on the upstream receptors in the monocyte‐to‐M2 differentiation, and the corresponding ligand expressed in other cell types. For this analysis, we utilized a comprehensive ligand‐receptor database IUPHAR (International Union of Pharmacology) and the Connectome^30^ database, which together encompass 790 ligands, 711 receptors, and 2,695 ligand‐receptor interactions.

**Figure 5:**
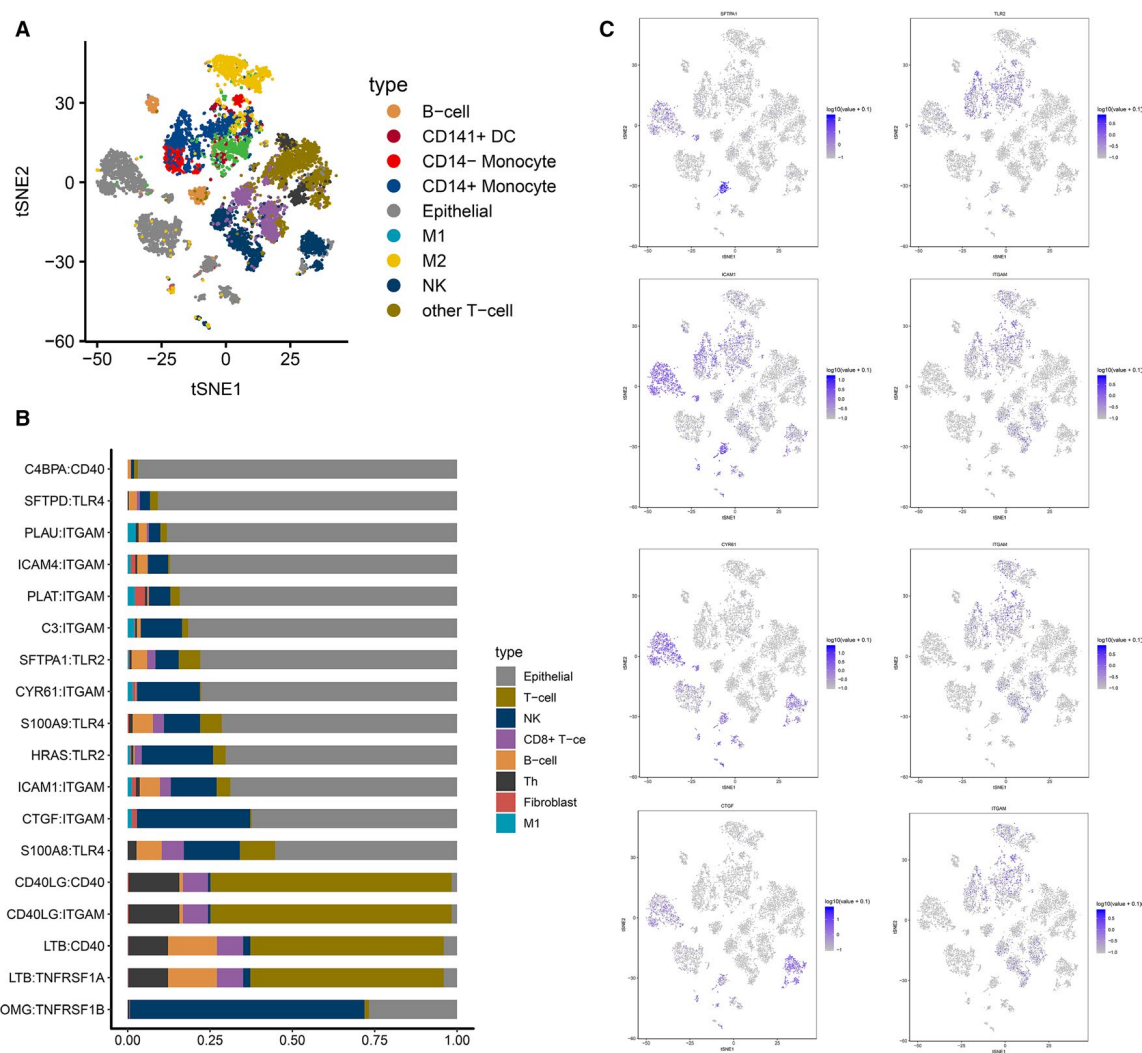
Intercellular interactions mediate the monocyte‐to‐M2 differentiation. (A). All single cells are visualized in the tSNE plot and are labeled by different colors to distinguish cell types. Epithelial cells, monocyte, and M2 macrophages are highlighted with shadows. (B). Bar plot depicts the percentage of ligand expressed by certain cell types, corresponding to the cognate receptors expressed by monocytes and M2 macrophages. The number of ligands was calculated based on their expression in scRNA‐seq data. All interactions refer to the specific indicated ligands from other cells that interact with the corresponding receptors expressed by monocytes and M2 macrophages. (C). The tSNE visualization of cells expressing ligands and corresponding receptors

The analysis identified 10 receptors on monocytes and M2 macrophages, and 30 cognate ligands expressed by other types of cells (Figure 5A,B). We found that epithelial cells expressed notably more ligands for these receptors than any other cell type, suggesting a predominating cross‐talk between epithelial and myeloid cells during the monocyte‐to‐M2 differentiation. Specifically, the identified interaction signals included the ligand‐receptor pairs *SFTPA1*‐*TLR2*, *ICAM1*‐*ITGAM*, *CYR61*‐*ITGAM*, and *CTGF*‐*ITGAM* (Figure 5B). From the expression patterns of these ligands and receptors (Figure 5C), we observed that only specific clusters of epithelial cells expressed these ligands, suggesting two functionally different subsets of epithelial cells that may impact monocyte‐to‐M2 signaling.

### 3.6 Intratumoral epithelial cells may impact the monocyte‐to‐M2 differentiation

**Figure 6:**
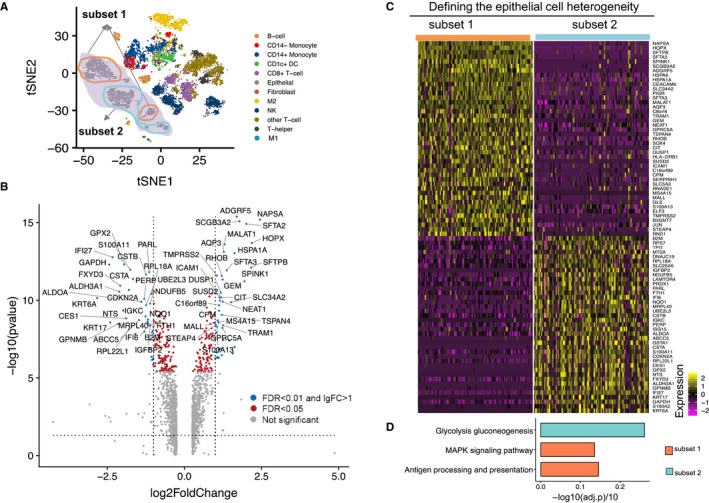
Intratumoral epithelial cells may impact the monocyte‐to‐M2 differentiation. (A). The t‐SNE plot highlights the epithelial cell subsets with shadows. (B). Volcano plot shows the differentially expressed genes between subset 1 versus subset 2. Log (fold change) of genes between the 2 subsets is plotted on the x‐axis, and the adjusted *P*‐value (−1 × log 10 scale) is plotted on the *y*‐axis. Red dots represent genes with adjusted *P*‐value < 0.05. Blue dots represent the genes with adjusted *P*‐value < 0.01 and |log2 FC|>1. (C). Heatmap shows the expression of the differentially expressed genes in the 2 epithelial cell subsets. Color scheme is based on z‐score distribution, from − 2 (purple) to 2 (yellow). Genes (rows) with (log2 Fold Change) >1 and adjusted *P*‐value < 0.01 are listed in respective of each subset. (D). Significantly, enriched pathways in the KEGG database (*y*‐axis) are shown in bar plots for each subset. The x‐axis represents ‐lg(adj.p)/10, which is calculated by the enrichment test (see Methods)

Using two subsets of epithelial cells (Figure 6A), that is, subset 1 (the 2 epithelial cell clusters expressing all these ligands including *SFTPA1*, *ICAM1*, *CTGF*, and *CYR61*) and subset 2 (the rest of epithelial cells), we assessed the tissue source and found that subset 1 was mostly (about 81%) from the tumor tissues, whereas subset 2 was mostly (about 91%) from the adjacent normal tissues (Figure S8B). Consequently, subset 1 consisted of intratumoral epithelial cells, whereas subset 2 consisted of mostly normal epithelial cells. To further characterize the 2 subsets, we performed differential expression analysis between the 2 subsets using DEseq2. We identified 43 upregulated genes and 40 downregulated genes (Figure 6B,C). Interestingly, oncogenes such as *JUN*, *MALAT1,* and *TMPRSS2* were identified as upregulated in the intratumoral epithelial cell subset (subset 1) compared to the normal epithelial cell subset (subset 2). Differentiation markers, that is, *CEACAM6* and *ICAM1*, were also upregulated in intratumoral epithelial cell subset. Interestingly, *HLA‐DRB1* was highly expressed in the intratumoral epithelial cell subset.

A comprehensive list of the significantly differentially expressed genes is provided in Table S5. Enrichment analysis using the KEGG pathway databases (Figure 6D) showed that the genes over‐expressed in subset 1 were associated with *MAPK* signaling and the antigen processing and presentation pathways. In contrast, the genes highly expressed in subset 2 were associated with the glycolysis gluconeogenesis pathway. These results highlighted the different functional property of subset 1 (ie, intratumoral epithelial cells), and their potential impact of the monocyte‐to‐M2 differentiation.

## 4 DISCUSSION

The lung is constantly exposed to an array of foreign substances. As a result, this tissue experiences high immunologic activity that requires responsiveness to potentially dangerous pathogens. Carcinogens from smoking and environmental pollutants cause mutational events in epithelial cells, suppress immune surveillance, and impact the efficacy of anti‐PD‐1 therapy.^41^ Development of immune checkpoint inhibitor therapies has led to a durable response in 20%‐30% of advanced NSCLC. Furthermore, a recent study using neoadjuvant immunotherapy showed that immune checkpoint inhibitors resulted in significant responses in 9 of 20 early‐stage NSCLC cases,^5^ suggesting that the immunosuppression that exists in advanced NSCLC is already in place at earlier stages of lung cancer. This observation prompted us to survey the tumor‐infiltrating immune landscape in early stage NSCLC. To this end, we performed scRNA‐seq analysis with fresh surgical samples from 4 NSCLC patients. By comparative analysis of matched tumor and normal tissue specimens, we observed extensive differences in the proportions and transcriptional architectures of both lymphoid and myeloid cell compartments. CD8 + cytotoxic T cells and NK cells were commonly decreased in tumor relative to adjacent normal tissue, a biological state consistent with immune evasion that likely favors tumor cell survival and growth. We also observed a consistent myeloid cell presence in normal and tumor specimens that, from a transcriptional perspective, varied directionally from normal to tumor tissues, consistent with a protumorigenic monocyte‐to‐M2 polarization trajectory. Of note, though the trajectory based on the single‐cell RNA‐seq data of myeloid cells determined a developmental ordering (trajectory) of cells without any time‐point experiments, our analysis provides a quantitative, high‐resolution ordering of surface marker expression, and signaling events. Such precise ordering of key events explicitly pinpointed the developmental hallmarks of myeloid cell reprogramming.

As a significant component of intratumoral myeloid cells, M2 macrophages are major contributors to the immunosuppressed TME. These macrophages express immunosuppressive cytokines and ligands that antagonize T‐cell mediated immunity, and they secrete chemokines and growth factors that recruit other protumorigenic immune cell populations (eg, T‐regs, TAMs, MDSCs) and directly induce cancer cell growth and spread through ligand‐receptor interactions. Therapeutic strategies to inhibit M2 macrophage differentiation and/or function are likely to have significant anti‐cancer activity. Emerging therapies currently employed in clinical trials (PLX3397: NCT02584647, NCT02071940, and NCT02371369; BLZ945: NCT02829723)^42^ target the CSF1R signaling axis and include the humanized monoclonal antibody, emactuzumab, and a variety of CSF1R kinase inhibitors (eg, BLZ945 and PLX3397). BLZ945 has been shown to block tumor progression in a glioblastoma multiforme (GBM) mouse model, human GBM xenografts,^43^ and murine models of cervical and mammary carcinomas^44^ suggesting potential pan‐cancer applications. However, prolonged CSF1R blockade can lead to acquired resistance through mechanisms associated with myeloid differentiation and molecular reprogramming in the TME. Specifically, T‐cell mediated IL‐4 production following CSF1R inhibition induces macrophage‐mediated secretion of IGF‐1 that in turn activates intratumoral PI3K signaling (via glioma‐expressed IGF‐1 receptor) and the subsequent survival and growth of glioma cells.^45^ Furthermore, this mechanism of drug resistance was subsequently validated and quantitatively described through spatio‐temporal mathematical modeling using input from molecular networks involved in myeloid‐glioma crosstalk signaling.^46^, ^47^ Our findings shed new light on the molecular nodes and signaling processes that underlie monocytes‐to‐M2 differentiation and myeloid‐tumor crosstalk, and provides a better understanding of myeloid‐mediated drug resistance mechanisms and facilitates the design of future immunotherapy strategies.

Infiltrating immune cells are key components of the TME. Mutagenic events in tumor cells can trigger a cascade of changes to the immune cellular ecosystem. We investigated this crosstalk and interaction using two approaches. First, the trajectory analysis revealed the monocyte‐to‐M2 transition within the myeloid cell population, along with molecular changes as evidenced by the upregulation of M2‐macrophage marker genes (*MRC1/CD206, MSR1/CD204, PPARG, *and *TREM2*). This analysis also showed that the upregulation of *ITGAM*, a driver of M2 polarization, promotes *STAT6* activation via *IL‐13* and *IL‐4* signaling.^48^ Additionally, we identified JUN as an upstream transcription factor regulating monocyte‐to‐M2 differentiation, suggesting the potential role of JUN in myeloid cell reprogramming. This discovery aligns with previous reports suggesting a role for JUN in monocyte and macrophage differentiation.^49^, ^50^ Second, the single cell RNA‐seq approach provided a unique opportunity to identify physiologically relevant tumor derived signals. We identified tumor‐derived ligands that may mediate the monocyte‐to‐M2 differentiation. For example, our analysis identified intratumoral epithelial expression of *ICAM1*,^52^ the ligand for the receptor *ITGAM* which is expressed on the surface of monocytes and M2 macrophages, suggesting a role of intratumoral epithelial cells in regulating the monocyte‐to‐M2 differentiation in the TME.

The differentiation from monocytes to macrophages is mediated by TME signals. Monocytes, known for their functional and molecular plasticity, can be recruited to the tumor site by tumor‐secreted chemo‐attractants (eg, CCL2, S100A8/S100A9), and polarized toward M1 or M2 macrophages depending on the cytokine milieu. Our trajectory analysis across patients revealed markers of the process from monocyte recruitment to components of the transition from monocytes to M2 macrophages, but with little information concerning the role of M1‐polarized macrophages. In our study, M1 macrophages were rarely observed and their sparse presence limited a detailed analysis, consistent with significantly reduced levels of M1 cells as compared to M2 cells in NSCLC tumors.^53^

In summary, interrogation into the immune ecosystem in early stage NSCLC has revealed remarkable heterogeneity and plasticity in the myeloid compartment. Our findings support the idea that M2 macrophage activation is consistent with a differentiation model, either as discrete states or along a spectrum of alternative trajectories. Since current immune checkpoint inhibitor therapies exhibit limited efficacy in some patients, our observations that myeloid cells are highly reprogrammed toward protumor M2 macrophages offer another possible therapeutic opportunity in the arena of immunotherapy.

## CONFLICT OF INTEREST

The authors declare that they have no competing interests.

## AUTHORS’ CONTRIBUTIONS

WZ and LDM conceived of and designed the study. LW, EL, GK, and SO facilitated tissue acquisition via TTPSR and contributed to the analysis of the data. EF isolated single cells from surgical samples, with input from LDM and WZ, and prepared samples for scRNA‐seq performed by the CGSR, with help from LC and under the supervision of GAH and LDM with additional support from WZ and GJ. QS performed the bioinformatics analysis, under supervision of WZ. QS, PC, WZ, and LDM wrote the manuscript, with input from all authors. All authors read and approved the final manuscript.

## Data Availability

The single cell RNA‐seq data are available in the NCBI Gene expression Omnibus database (GEO) with accession number GSE117570.
