# Supplementary material for: Dissecting intratumoral myeloid cell plasticity by single cell RNA‐seq
Source: Cancer Med. 2019 Apr 29;8(6):3072–85. doi: 10.1002/cam4.2113 (PMC6558497; doi:10.1002/cam4.2113)
Supplement: Supplementary file 1 [file CAM4-8-3072-s001.pdf]

Figure S1

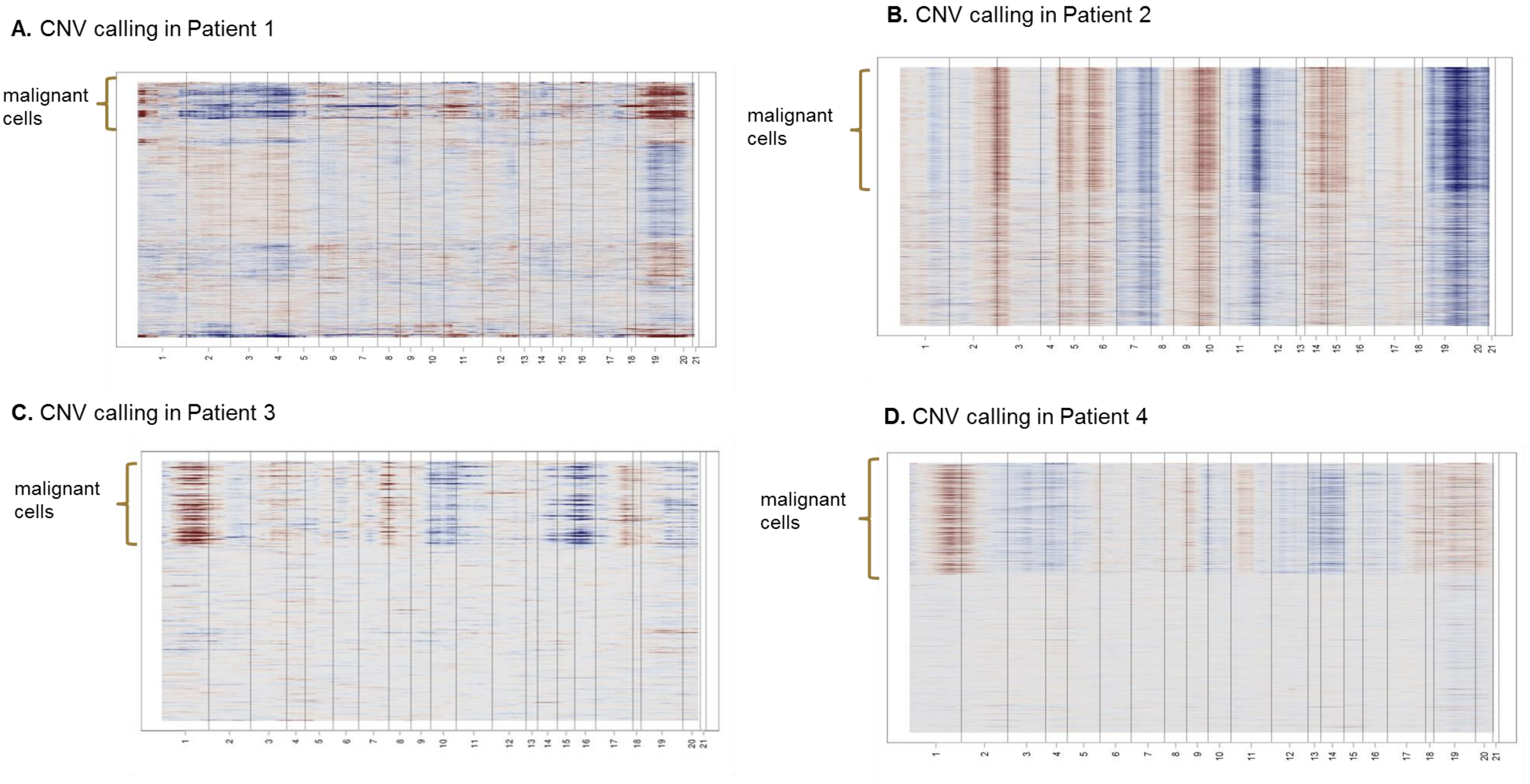

(A-D). Visualization of chromosomal alterations in cells (rows) along the genome (columns) for each patient. Colors indicate copy number gains (red) and losses (blue). Expression in each cell was smoothed in a sliding window along genes ordered by their chromosomal location. The average expression in non-malignant cells was used to normalize the expression data. Therefore, non-malignant cells, are the ones without alterations. The legend represents the visualization threshold. In each patient, the cells with copy number alterations have much overlap with our annotated epithelial cell clusters with enriched expressions of epithelial markers. The overlapping percentages are respectively P1, 84.2%; P2, 79%; P3, 88%; P4, 81%. In our study, we remove these cells in overlap or with enriched epithelial markers for further analysis.

Figure S2

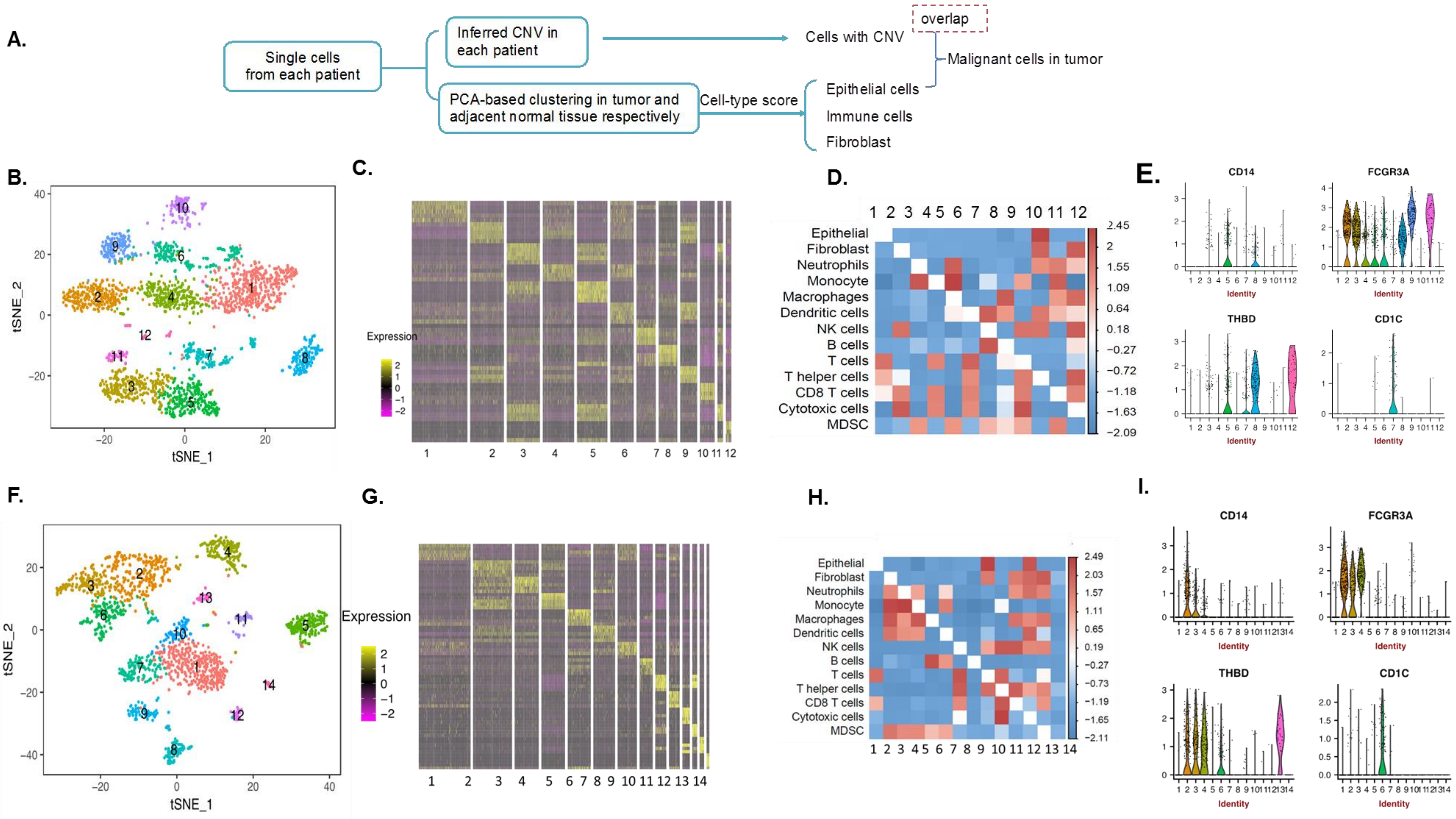

(A) The annotation workflow shows our major steps for single cell annotation in each patient.
(B) The t-Distributed Stochastic Neighbor Embedding (t-SNE) visualization of single cells from the adjacent normal tissues of Patient 1. Single cells were clustered to 12 clusters labeled from 1 to 12 respectively. Cell clusters are represented by different colors..
(C) Heatmap shows the expression pattern of top five differential genes in each cell cluster.
(D) The graph shows the value of log10(cell-type score) for the 12 cell clusters. Rows represent the cell clusters and columns represent the cell types.
(E) Violin plots show the expression level distributions of specific genes (i.e. CD14, CD16, CD1c, CD141) across cell clusters. Cell clusters are represented by different colors.
(F) The tSNE visualization of single cells from the tumor tissues of Patient 1. Single cells were clustered to 14 clusters labeled from 1 to 14 respectively. Cell clusters are represented by different colors.
(G) Heatmap shows the expression pattern of top five differential genes in each cell cluster.
(H) The graph shows the value of log10(cell-type score) for the 14 cell clusters. Rows represent the cell clusters and columns represent the cell types.
(I) Violin plots show the expression level distributions of specific genes (i.e. CD14, CD16, CD1c, CD141) across cell clusters. Cell clusters are represented by different colors.

Figure S3

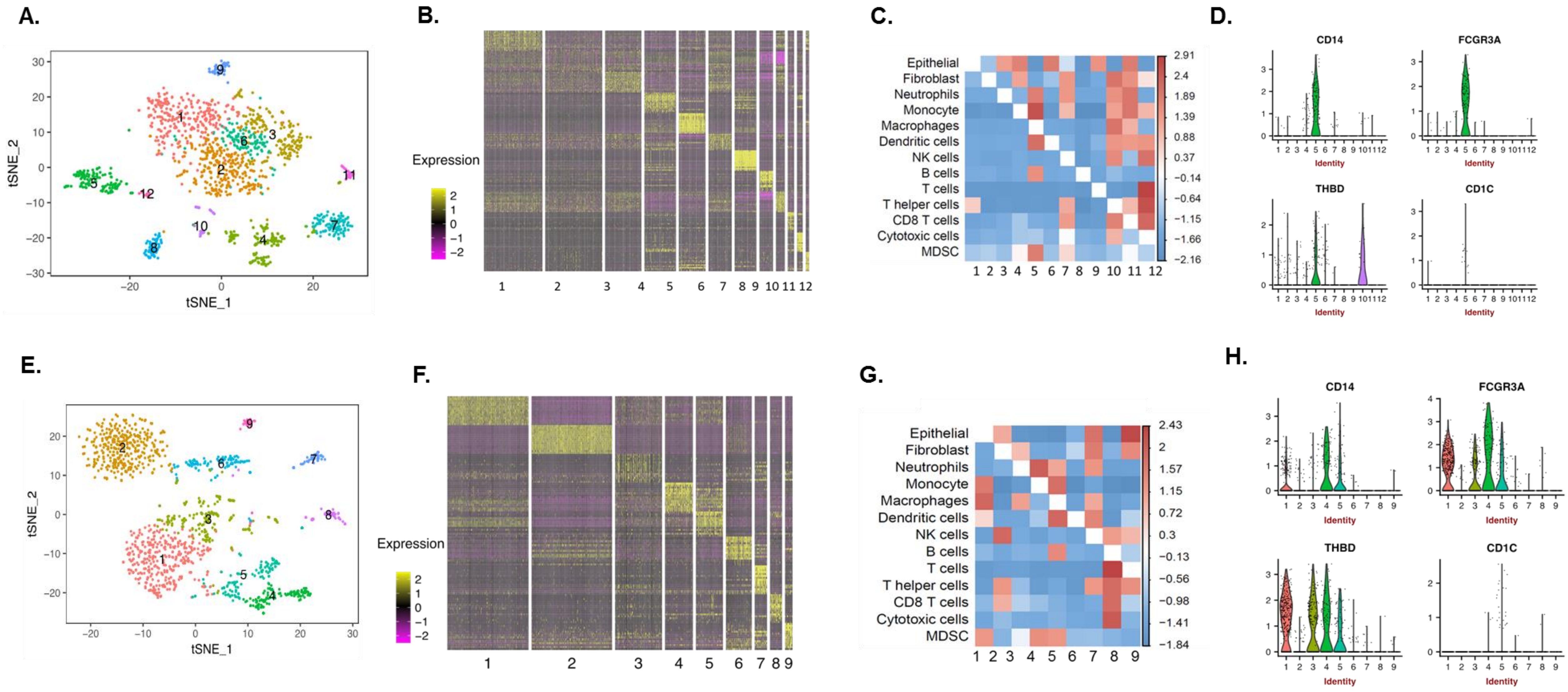

(A) The annotation workflow shows our major steps for single cell annotation in each patient.
(B) The t-Distributed Stochastic Neighbor Embedding (t-SNE) visualization of single cells from the adjacent normal tissues of Patient 2. Single cells were clustered to 12 clusters labeled from 1 to 12 respectively. Cell clusters are represented by different colors..
(C) Heatmap shows the expression pattern of top five differential genes in each cell cluster.
(D) The graph shows the value of log10(cell-type score) for the 12 cell clusters. Rows represent the cell clusters and columns represent the cell types.
(E) Violin plots show the expression level distributions of specific genes (i.e. CD14, CD16, CD1c, CD141) across cell clusters. Cell clusters are represented by different colors.
(F) The tSNE visualization of single cells from the tumor tissues of Patient 2. Single cells were clustered to 14 clusters labeled from 1 to 9 respectively. Cell clusters are represented by different colors.
(G) Heatmap shows the expression pattern of top five differential genes in each cell cluster.
(H) The graph shows the value of log10(cell-type score) for the 9 cell clusters. Rows represent the cell clusters and columns represent the cell types.
(I) Violin plots show the expression level distributions of specific genes (i.e. CD14, CD16, CD1c, CD141) across cell clusters. Cell clusters are represented by different colors.

Figure S4

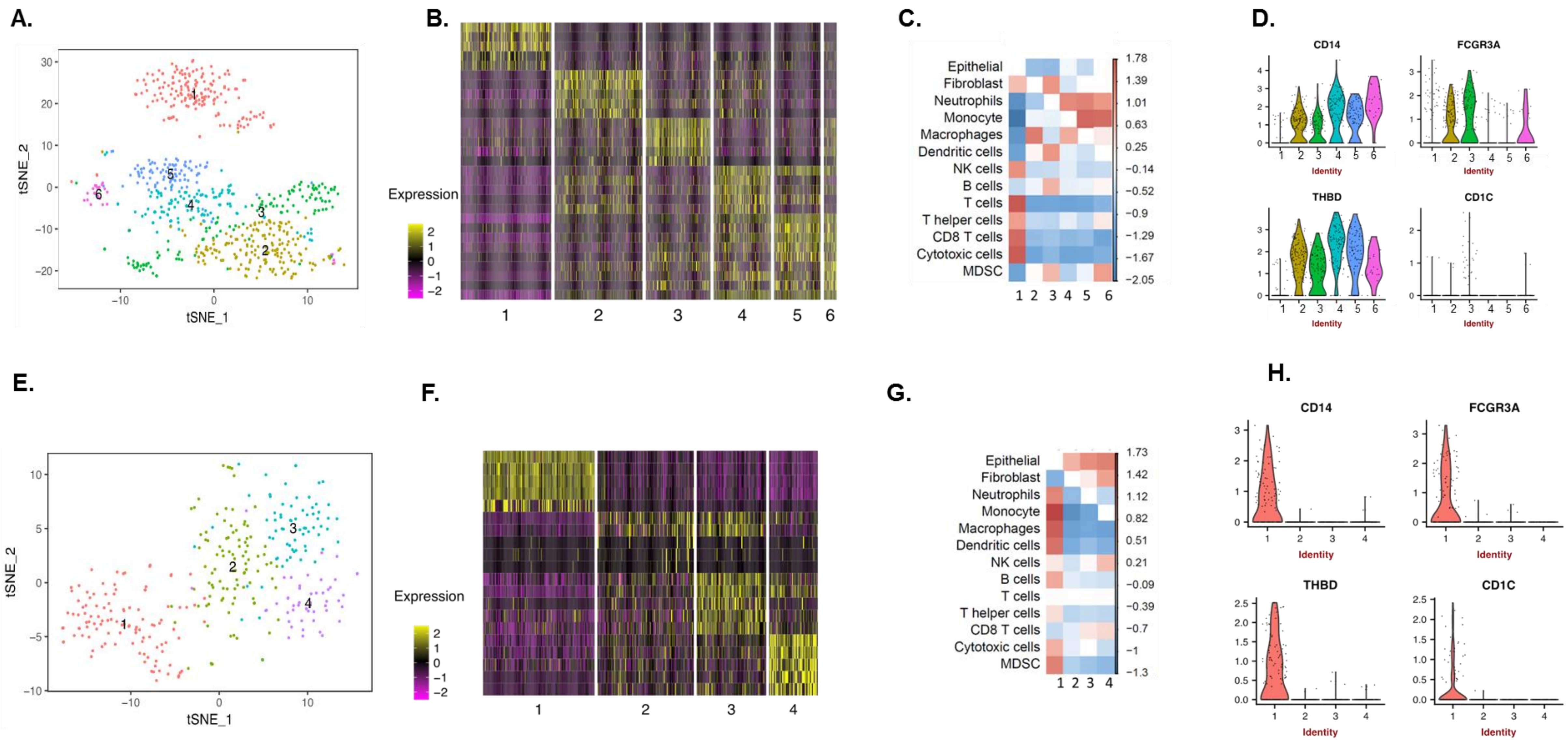

(A) The annotation workflow shows our major steps for single cell annotation in each patient.
(B) The t-Distributed Stochastic Neighbor Embedding (t-SNE) visualization of single cells from the adjacent normal tissues of Patient 3. Single cells were clustered to 6 clusters labeled from 1 to 12 respectively. Cell clusters are represented by different colors..
(C) Heatmap shows the expression pattern of top five differential genes in each cell cluster.
(D) The graph shows the value of log10(cell-type score) for the 6 cell clusters. Rows represent the cell clusters and columns represent the cell types.
(E) Violin plots show the expression level distributions of specific genes (i.e. CD14, CD16, CD1c, CD141) across cell clusters. Cell clusters are represented by different colors.
(F) The tSNE visualization of single cells from the tumor tissues of Patient 3. Single cells were clustered to 14 clusters labeled from 1 to 4 respectively. Cell clusters are represented by different colors.
(G) Heatmap shows the expression pattern of top five differential genes in each cell cluster.
(H) The graph shows the value of log10(cell-type score) for the 4 cell clusters. Rows represent the cell clusters and columns represent the cell types.
(I) Violin plots show the expression level distributions of specific genes (i.e. CD14, CD16, CD1c, CD141) across cell clusters. Cell clusters are represented by different colors.

Figure S5

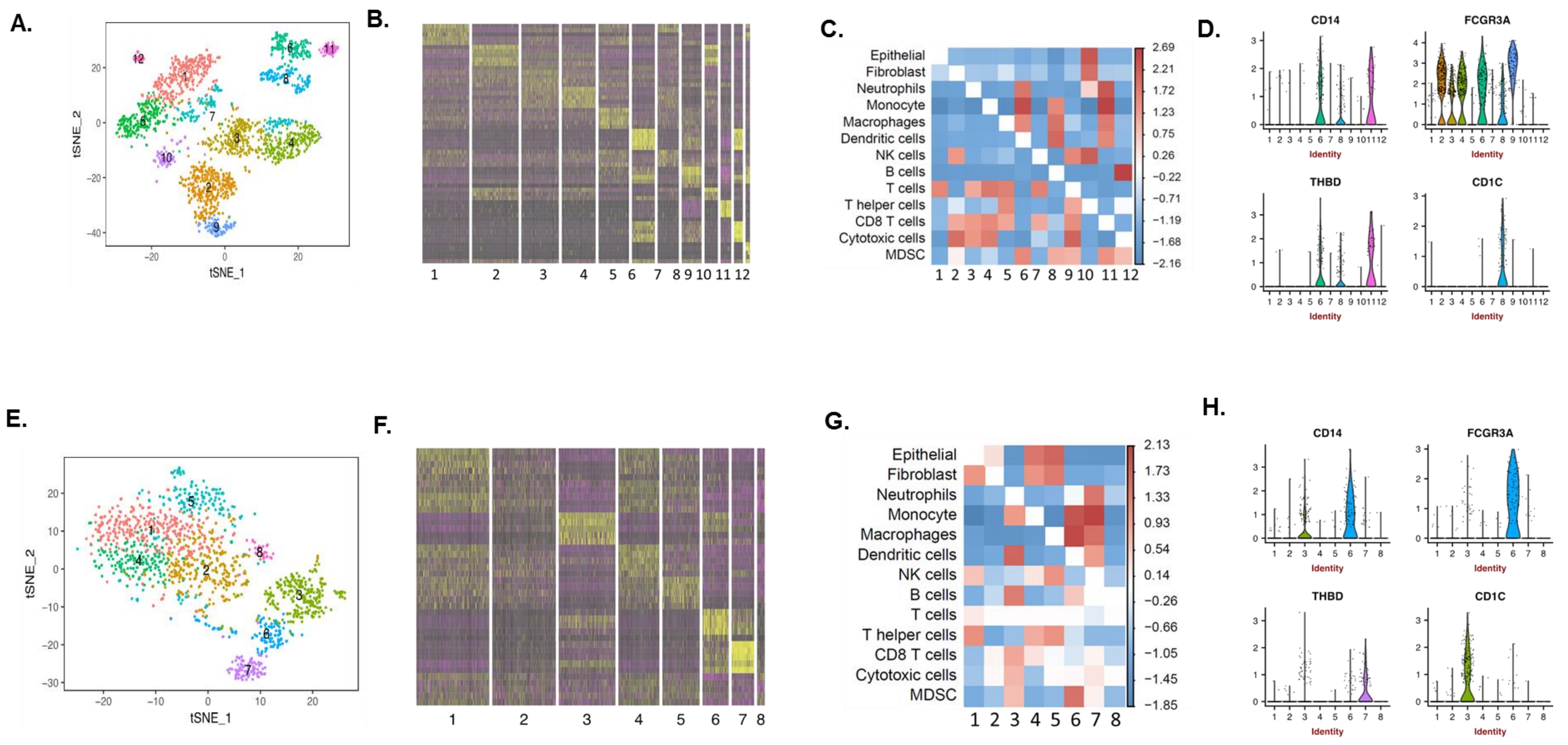

(A) The annotation workflow shows our major steps for single cell annotation in each patient.
(B) The t-Distributed Stochastic Neighbor Embedding (t-SNE) visualization of single cells from the adjacent normal tissues of Patient 4. Single cells were clustered to 12 clusters labeled from 1 to 12 respectively. Cell clusters are represented by different colors..
(C) Heatmap shows the expression pattern of top five differential genes in each cell cluster.
(D) The graph shows the value of log10(cell-type score) for the 12 cell clusters. Rows represent the cell clusters and columns represent the cell types.
(E) Violin plots show the expression level distributions of specific genes (i.e. CD14, CD16, CD1c, CD141) across cell clusters. Cell clusters are represented by different colors.
(F) The tSNE visualization of single cells from the tumor tissues of Patient 4. Single cells were clustered to 14 clusters labeled from 1 to 8 respectively. Cell clusters are represented by different colors.
(G) Heatmap shows the expression pattern of top five differential genes in each cell cluster.
(H) The graph shows the value of log10(cell-type score) for the 8 cell clusters. Rows represent the cell clusters and columns represent the cell types.
(I) Violin plots show the expression level distributions of specific genes (i.e. CD14, CD16, CD1c, CD141) across cell clusters. Cell clusters are represented by different colors.

Figure S6

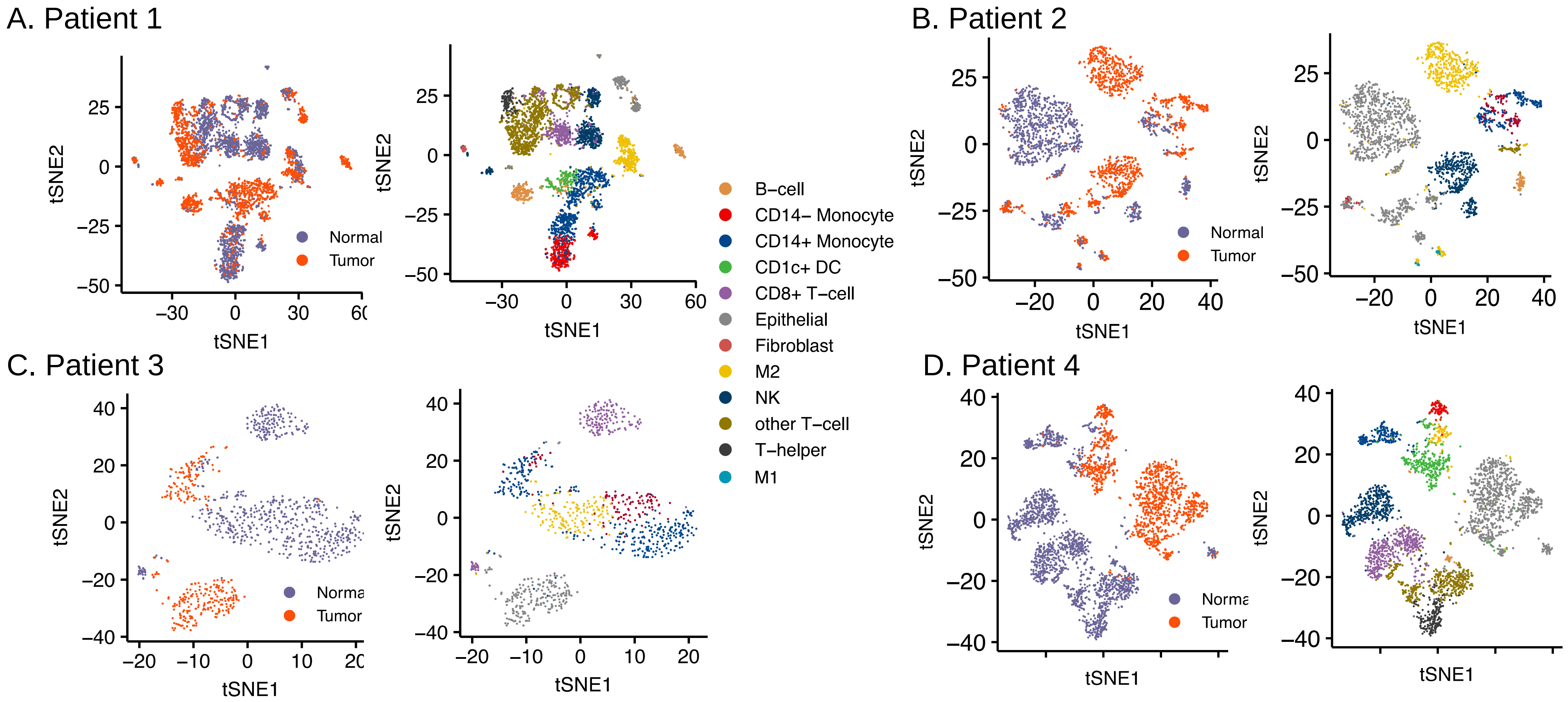

(A-D). Each plot illustrates single cells from an individual patient (P1-P4). For each patient, the left panel depicts the tSNE visualization of single cells labeled by color to distinguish their tissue source, i.e. adjacent normal tissue (cyan) or tumor tissue (orange). The right panel shows the same cells color-coded by their annotated cell types.

Figure S7

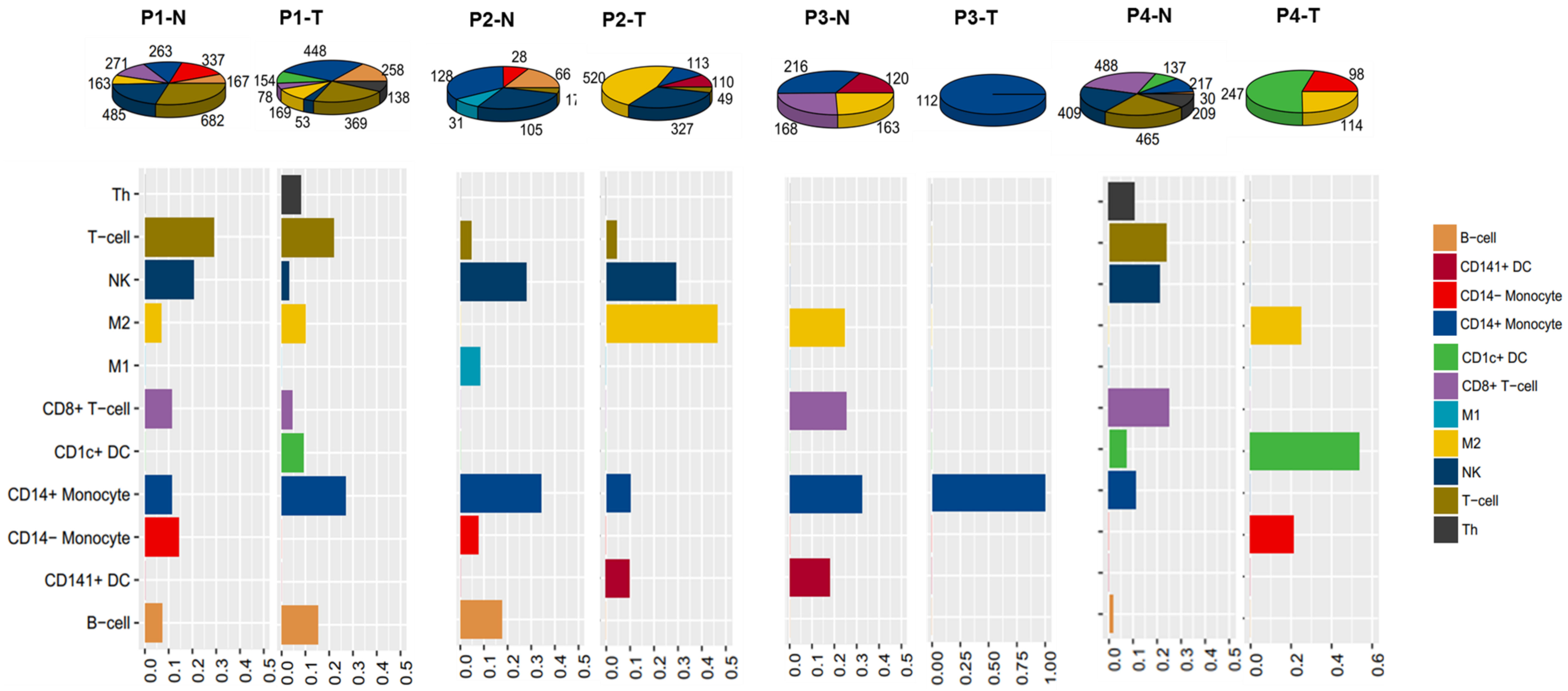

The upper panel in the plot shows the cell number of the annotated immune cells in both adjacent normal and tumor tissue of each patient. The lower panel in the plot shows the proportions of the annotated immune cells in both adjacent normal and tumor tissue of each patient.

Figure S8

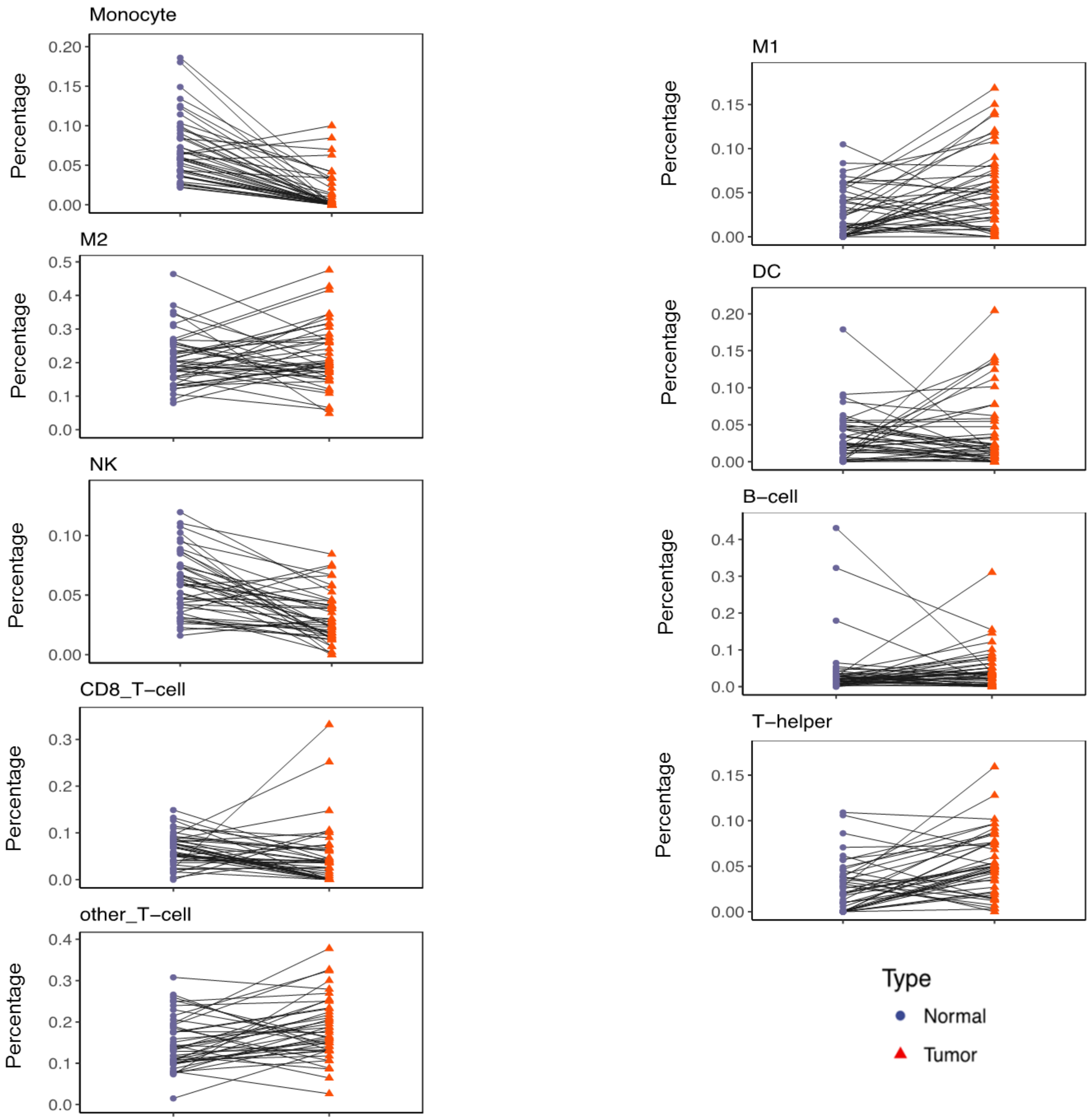

Line plots dictate the immune cell infiltrates in the adjacent normal and respective tumor samples of early-stage NSCLC patients from TCGA (n=88). Their relative fraction of 22 immune cell types was estimated using CIBERSORT tool (Cell-type Identification By Estimating Relative Subsets Of RNA Transcripts) (86). In order to relate to results to other estimates in this study, the aggregation schemes were defined as follows (8 classes). We take the patients with estimated p-value (by CIBERSORT) <0.05, only 44 paired patients left.
Monocytes = Monocytes
M1 macrophages = Macrophages M1
M2 macrophages = Macrophages M2,
DC = Dendritic cells resting + Dendritic cells activated
CD8+ T-cells = T cells CD8
T-helper cells = T cells follicular helper
Other T-cells = T cells CD4 naïve + T cells CD4 memory resting + T cells CD4 memory activated + T cells regulatory (Tregs) + T cells gamma delta
NK = NK cells resting + NK cells activated
B-cells = B cells naive + B cells memory

Figure S9

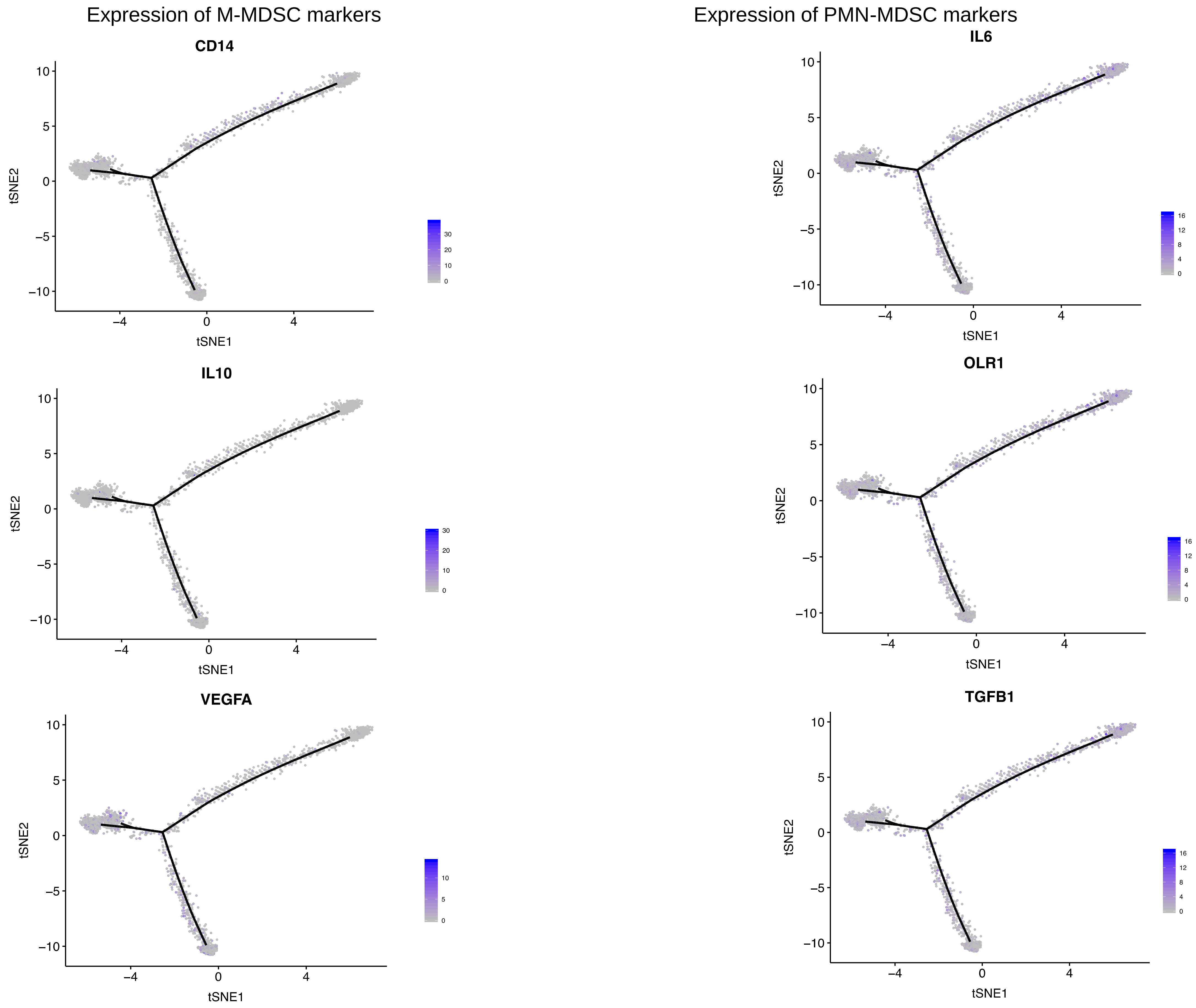

t-SNE of single cells from each patient, colored by the expression of M-MDSC and PMN-MDSC markers that were identified in the single cell data.

Figure S10

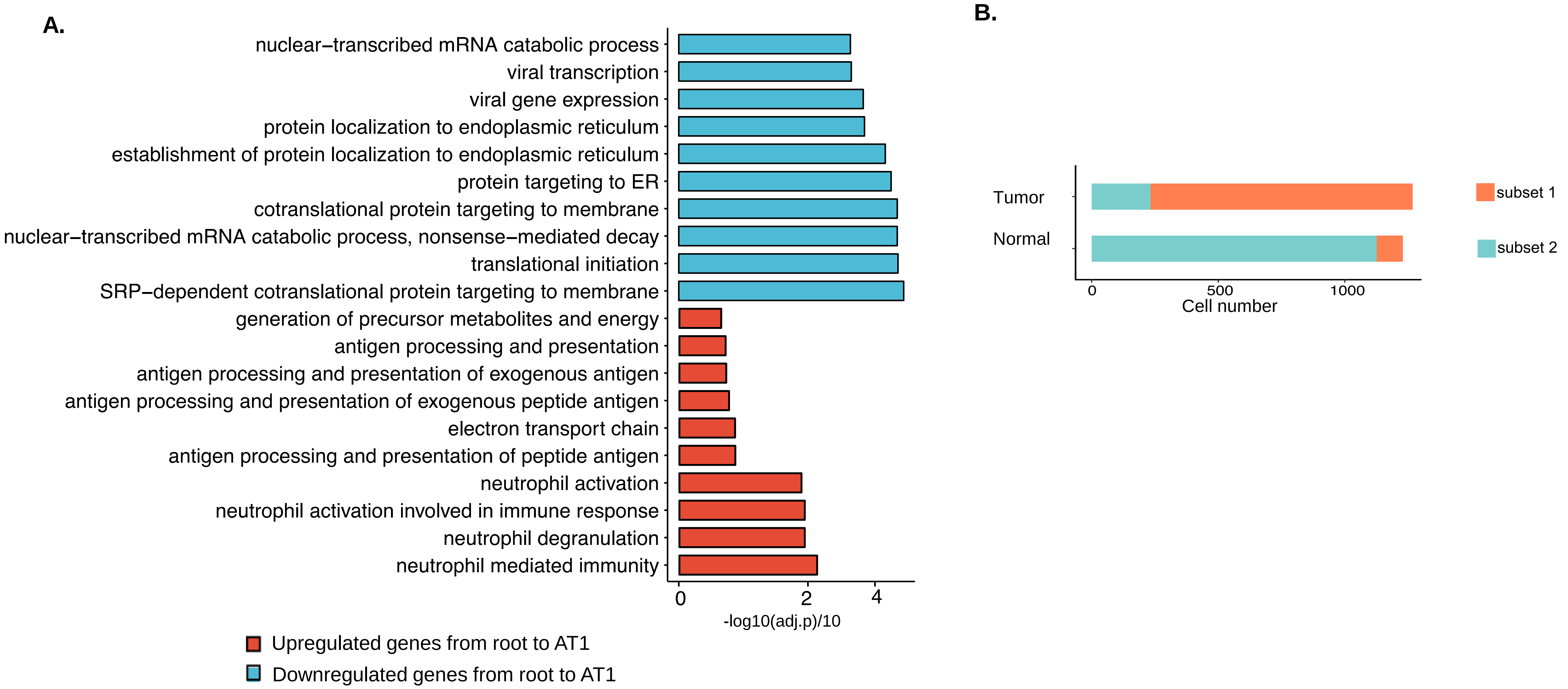

(A). Bar plots show the significant enriched GO biological terms for these upregulated and downregulated genes along the differentiation from monocytes to M2 macrophages. The x-axis represents -log10(adj.p-value)/10 as described in Method. (B). Barplots show the fraction of different epithelial subsets from the tumor or adjacent normal tissue.

Additional File 2: Table S1 Single cell specimen information. Table S2 Quality control. Table S3 Cell type specific markers. Table S4 Hub JUN encompassing genes. Table S5 Differential expressed genes between two subsets of epithelial cells.
